# Linking late cognitive outcome with glioma surgery location using resection cavity maps

**DOI:** 10.1002/hbm.23986

**Published:** 2018-01-29

**Authors:** Eef J. Hendriks, Esther J. J. Habets, Martin J. B. Taphoorn, Linda Douw, Aeilko H. Zwinderman, W. Peter Vandertop, Frederik Barkhof, Martin Klein, Philip C. De Witt Hamer

**Affiliations:** ^1^ Department of Radiology & Nuclear Medicine VU University Medical Center Amsterdam The Netherlands; ^2^ Department of Neurology Medical Center Haaglanden The Hague The Netherlands; ^3^ Department of Neurology Leiden University Medical Center Leiden The Netherlands; ^4^ Department of Anatomy and Neurosciences VU University Medical Center Amsterdam The Netherlands; ^5^ Department of Radiology Athinoula Martinos Center for Biomedical Imaging, Massachusetts General Hospital Charlestown Massachusetts; ^6^ Department of Clinical Epidemiology and Biostatistics Academic Medical Center Amsterdam The Netherlands; ^7^ Neurosurgical Center Amsterdam, VU University Medical Center and Academic Medical Center Amsterdam The Netherlands; ^8^ Institutes of Neurology & Healthcare Engineering, UCL London United Kingdom; ^9^ Medical Neuropsychology Section, Department of Medical Psychology VU University Medical Center Amsterdam The Netherlands

**Keywords:** cognition, glioma, magnetic resonance imaging, neurosurgery

## Abstract

Patients with a diffuse glioma may experience cognitive decline or improvement upon resective surgery. To examine the impact of glioma location, cognitive alteration after glioma surgery was quantified and related to voxel‐based resection probability maps. A total of 59 consecutive patients (range 18–67 years of age) who had resective surgery between 2006 and 2011 for a supratentorial nonenhancing diffuse glioma (grade I–III, WHO 2007) were included in this observational cohort study. Standardized neuropsychological examination and MRI were obtained before and after surgery. Intraoperative stimulation mapping guided resections towards neurological functions (language, sensorimotor function, and visual fields). Maps of resected regions were constructed in standard space. These resection cavity maps were compared between patients with and without new cognitive deficits (*z*‐score difference >1.5 *SD* between baseline and one year after resection), using a voxel‐wise randomization test and calculation of false discovery rates. Brain regions significantly associated with cognitive decline were classified in standard cortical and subcortical anatomy. Cognitive improvement in any domain occurred in 10 (17%) patients, cognitive decline in any domain in 25 (42%), and decline in more than one domain in 10 (17%). The most frequently affected subdomains were attention in 10 (17%) patients and information processing speed in 9 (15%). Resection regions associated with decline in more than one domain were predominantly located in the right hemisphere. For attention decline, no specific region could be identified. For decline in information speed, several regions were found, including the frontal pole and the corpus callosum. Cognitive decline after resective surgery of diffuse glioma is prevalent, in particular, in patients with a tumor located in the right hemisphere without cognitive function mapping.

## 1 INTRODUCTION

Standard care for diffuse glioma of grade WHO I–III includes resective surgery, followed by radio‐ and/or chemotherapy, depending on tumor grade, subtype, and molecular markers, either adjuvant or delayed at recurrence. The incidence of diffuse glioma is 5 per 100,000; nearly half are of WHO grade I–III (Ostrom et al., 2015). These tumors infiltrate the brain, recur, transform into higher grade gliomas, and are eventually lethal (Ricard et al., 2012).

Glioma surgery aims to improve survival by maximizing tumor removal (Capelle et al., 2013; Jakola et al., 2012), while preserving integrity of brain function (De Witt Hamer, Robles, Zwinderman, Duffau, & Berger, 2012). Nevertheless, glioma resections in general result in permanent neurological deficits in 7.1% of patients, mainly, motor and language dysfunction, which is 3.4% when neurological function mapping is applied (De Witt Hamer et al., 2012). Cognitive outcome after glioma surgery has been less well studied, mainly focused on short‐term outcome in WHO grade IV glioma with diverging results (Klein, 2012; Satoer et al., 2014; Talacchi, Santini, Savazzi, & Gerosa, 2011). Cognitive assessment in patients with brain tumors so far has demonstrated several factors contributing to cognitive functioning, such as tumor effects, seizures, medication, and oncological treatment (Douw et al., 2009; Klein, 2012). Cognitive functioning is of particular relevance for those patients diagnosed with WHO grade I–III gliomas, who are typically young adults with an anticipated survival of many years. Cognitive performance is important for social and professional functioning during these years. Consequently, here we focus on this population aiming to identify the relation between glioma surgery regions and late cognitive outcome. Knowledge on the relation between cognitive functioning and the resection site is important for patient counseling and surgical decision making.

Inherent to glioma resection is a locationalistic perspective on the brain: that some regions are more salient than others, surgical damage of which may induce brain dysfunction. At the same time, however, a more comprehensive framework consists of dynamic networks connected in parallel, and to which network theory applies (De Benedictis & Duffau, 2011). Several networks have been identified as relevant for cognitive functioning, and particular regions that are centrally located in the brain network, the so‐called hubs (Buckner et al., 2009; Cole et al., 2013; Power, Schlaggar, Lessov‐Schlaggar, & Petersen, 2013). This framework may be important for surgical treatment to understand potential loss of brain function.

We set out to increase our understanding of cognitive decline after glioma surgery by pinpointing resected regions that are shared among patients with cognitive decline using the well‐established method of lesion‐function mapping, which has not been applied to this knowledge gap in glioma resections. Here we aim to quantitate late cognitive outcome after glioma surgery and to link cognitive decline with resected regions.

## 2 METHODS

### 2.1 Patients

An observational cohort of consecutive patients at our tertiary referral center for neuro‐oncological care was identified. Standardized cognitive assessment and MR imaging were obtained as part of routine care.

Approval of the study protocol by the institutional review board (VU University Medical Center, Medical Ethical Research Committee, Amsterdam, The Netherlands) was not required according to the Dutch law on Medical Research in Humans. All patients provided informed consent for use of their clinical data for medical research. Data and imaging were analyzed after anonymization in accordance with the Personal Data Protection Act and the Code of conduct for responsible use of Human Tissue and Medical Research.

Patients were included, who (a) were over 17 years of age, (b) were diagnosed with a supratentorial glioma of grade I–II, or III with focal anaplasia—WHO 2007, (c) had a resection between 1/1/2006 and 31/12/2011, (d) had no prior radiotherapy to avoid MRI signal misinterpretation, (e) had pre‐ and postoperative cognitive assessment in seven cognitive domains, (f) had a pre‐ and postoperative MRI available, and (g) had stable disease between surgery and postoperative assessment. Furthermore, patients with another neurological or psychiatric disorder or with insufficient Dutch language skills were excluded, as this could have interfered with cognitive assessment.

Patients received standard care, including complete neurological examination by neurologists and neurosurgeons at presentation and during follow‐up. Treatment decisions were made in consensus by experts in tumor board meetings. Preoperative MRIs included functional MRI and DTI when deemed necessary. A dedicated neurosurgical team applied intraoperative stimulation mapping under local anesthesia and neurophysiological monitoring using EMG and EEG corticography, as needed. A selection of neurological functions was mapped in 50 (85%) patients: language, sensorimotor function, visual fields, and line bisection. Cognitive functions were not mapped during surgery.

### 2.2 Cognitive assessment

Cognitive performance was quantified for each patient in seven domains before and after surgery using standardized neuropsychological tests, which are specific for cognitive tasks potentially affected in brain tumor patients and sensitive for detection over time (Table 1) (Klein et al., 2001; Meyers & Brown, 2006). These seven cognitive domain scores were based on previous studies and consensus in neuropsychological practice (Douw et al., 2009; Meyers & Cantor, 2003). For each test, the patient's score was compared to published normative data of healthy controls of similar age, gender, and educational level (Jolles, van Boxtel, Ponds, Metsemakers, & Houx, 1998; Kessels, Nys, Brands, van den Berg, & Van Zandvoort, 2006; Schmand, Groenink, & Dungen, 2008; Wechsler, 2000) to calculate *z*‐scores. Subsequently, a performance score was calculated for every patient in each of seven cognitive domains using a combination of specific test *z*‐scores (Table 1). A domain *z*‐score was obtained by dividing the summed *z*‐scores of the tests contributing to that domain, by the number of tests within the domain. For each patient, scores were acquired at two time‐points: baseline measurement one week prior to surgery and postoperative after one year to avoid measurements during rehabilitation. These two time‐points allowed calculation of alteration per cognitive domain for every patient by subtracting the follow‐up from the baseline measurement. A decrease in *z*‐score of more than 1.5 was defined as cognitive decline; an increase of more than 1.5 as cognitive improvement (Lezak, 2012). Hence, we classified each patient in every domain as cognitively stable, improved, or declined.

**Table 1:** Cognitive domains and tests

Cognitive domain	Cognitive test	Objective measure
Attention	Digit span Stroop color word test	Subtest: forward (number correct × span: 0–144)^a^ Subtests: card II: color condition, card III: color‐word condition (both time in seconds)^a^ andinterference^b^
Information speed	Stroop color word test Trailmaking Test Letter digit modalities test	Subtest: card I: word condition (time in seconds)^b^ Subtest: condition A (time in seconds)^b^ 90 seconds writing and reading (both number correct: 0–125)^a^
Visual construction	Rey complex figure test	Subtest: copy (number correct: 0–36)^a^
Verbal memory	Auditory verbal learning test	Total of five trials (number correct: 0–75)^a^ Delta score (number correct: 0–15)^a^ Delayed recall (number correct: 0–15)^a^ Delayed recognition (number correct: 0–30)^a^
Visual memory	Location learning test Rey complex figure test	Total of five trials (number incorrect: 0–∞)^b^ Learning index (score 0–1)^a^ Active delayed recall (number incorrect: 0–∞)^b^ Subtest: delayed recall (number correct: 0–36)^a^
Working memory	Digit span Memory comparison test	Subtest: backward (number correct × span: 0–112)^a^ Slope (time score)^b^ Intercept (time score)^b^
Execution	Trailmaking test Categoric word fluency test Letter word fluency test Behavioral assessment of the dysexecutive syndrome	Subtest: condition B (time in seconds); B/A^b^ Animals (number correct: 0–∞)^a^ Letters D, A, and T (numbers correct: 0–∞)^a^ Subtest: rule shifting test (profile score: 0–4)^a^

a Higher score means better performance;

b Higher score means worse performance.

To identify the patients with worst—most clinically relevant—cognitive performance in our analysis, we distinguished between those with more than one and those with at most one cognitive domain decline.

### 2.3 MR‐scanning

Patients were scanned before and after resective surgery using a standardized neuro‐oncology protocol. For analysis, we used the scans scheduled at 4 months postoperatively to avoid surgical artefacts. The protocol included a 3D FLAIR turbo spin‐echo pulse‐sequence and a 3D heavily T1‐weighted gradient‐echo pulse‐sequence obtained after double‐dose administration of intravenous gadolinium. MR scans were available from various 1.5 and 3.0 T scanners (including GE Signa HDXT, Toshiba Titan, Siemens Avanto, and Philips Ingenia). All MR images were acquired with ∼1 mm isotropic resolution.

### 2.4 Voxel‐based maps of resected regions

The resected regions were segmented in 3D on the postoperative FLAIR images (smart brush tool of iPlan v3.0 software; BrainLAB AG, Feldkirchen, Germany). The segmented volumes were verified and adjusted in reconstruction planes by two observers (EH and PW). The FLAIR scans were linearly registered to the T1 scans for further analysis using the image fusion tool. The segmented volumes were exported as binary masks registered to the T1 images. To bring these objects to MNI‐152 space (Fonov et al., 2009), the T1 images were nonlinearly registered in sequential steps, including rigid, affine, B‐spline regularization, and symmetric diffeomorphic registration with cross‐correlation as similarity metric (Avants, Epstein, Grossman, & Gee, 2008). The segmented objects were then aligned as binary images to MNI‐152 using these patient‐specific nonlinear transformations, resulting in maps of resected regions. Voxel‐based resection cavity maps of patient subgroups with and without cognitive decline could then be analyzed. The images and derived maps were downsampled to 2 × 2 × 2 mm for further analysis.

### 2.5 Statistical analysis to compare resection maps

To identify the brain regions that were associated with cognitive alteration upon resection, we set out to compare resection cavity maps of patients with and without a cognitive alteration using a voxel‐wise randomization test. We restricted the analysis to brain regions that were resected in at least 3 patients. As test statistic per voxel, we divided the fraction of patients with a resection region among those with an alteration by the fraction of patients with a resection region among those without, thus providing a “relative risk.” A higher test statistic corresponded to a higher rate of resection regions among patients with an alteration than among those without. To evaluate the probability of the test statistics, we used a randomization test. To estimate the empirical distribution under the null hypothesis of no difference by randomization, patient labels of cognitive alteration were randomized 4,000 times without replacement, as has been applied to analyze functional neuroimaging (Nichols & Holmes, 2002; Nichols & Hayasaka 2003; Schwartzman, Dougherty, Lee, Ghahremani, & Taylor, 2009). To obtain one‐sided *p* values per voxel, the number of equal or more extreme test statistics in the randomized null distribution were counted and divided by the number of randomizations. Next, to obtain *q* values per voxel to account for multiple testing, we divided the expected number of false‐discoveries based on the empirical null distribution by the number of discoveries across all voxels, if a voxel's *p* value would be declared significant (Langers, Jansen, & Backes, 2007; Nichols & Holmes 2002; Schwartzman et al., 2009). These *q* values can be interpreted as false discovery rates. Because of the exploratory nature of our research question, we deemed *q* values up to 40% of interest. Statistical procedures were customized in R (v3.3; R: A Language and Environment for Statistical Computing, R Core Team, R Foundation for Statistical Computing, Vienna, Austria, September, 2013). For visual inspection, the *p*‐ and *q*‐values were presented as overlays on MNI‐152. For further interpretation of the brain locations, we aligned our results with standard cortical anatomy (Desikan et al., 2006) and white matter anatomy (Catani & Thiebaut de Schotten, 2008).

## 3 RESULTS

### 3.1 Patient characteristics

Out of 68 eligible adults with a diffuse glioma, 9 were excluded due to prior therapy or early progression. The final cohort included 59 patients with a mean age of 39 (*SD* 11; range 18–67) years, of whom 25 (42%) were female. Patients presented with seizures in 54 (92%) or with an incidental finding in 5 (8%). At presentation language deficits were present in 13 (22%) and motor deficits in 5 (8%). Two (3%) patients were left‐handed.

The gliomas had an average volume of 71 (*SD* 67) mL and were located in the left hemisphere in 32 (54%). WHO grade II was diagnosed in 43 (73%, oligodendroglioma in 21, astrocytoma in 14, oligo‐astrocytoma in 8), grade III due to focal anaplasia in 10 (17%, anaplastic oligodendroglioma in 5, anaplastic astrocytoma in 4, anaplastic oligo‐astrocytoma in 1), and of grade I in 6 (10%, ganglioglioma in 4, pilocytic astrocytoma in 1, dysembryoplastic neuroepithelial tumor in 1).

The mean postoperative residual volume was 17 (SD 38) mL, and the mean extent of resection was 81% (*SD* 20%). Less than 10 mL residual tumor was observed in 35 (59%); more than 90% extent of resection was observed in 24 (41%). None of the patients had a permanent severe neurological deficit. In 5 (8%) a minor motor deficit of at least Medical Research Council grade 4 weakness was observed, in two (3%) a minor language deficit, dysnomia, was reported. Adjuvant radiotherapy was initiated in 13 (18%) and adjuvant temozolomide in 2 (3%).

Baseline performance in cognitive domains deviated from normative data (baseline *z*‐score <−1.5 *SD*) in 29 (49%) patients. Twelve (20%) patients deviated in one of seven domains. Seventeen (28%) deviated in more than one domain. Low scores at baseline were observed in 19 (32%) patients for attention, in 11 (19%) for working memory, in eight (14%) for verbal memory, in eight (14%) for visual memory, in six (10%) for information speed, in four (7%) for execution, and in one (2%) for visual construction.

### 3.2 Cognitive outcome per domain

**Figure 1:**
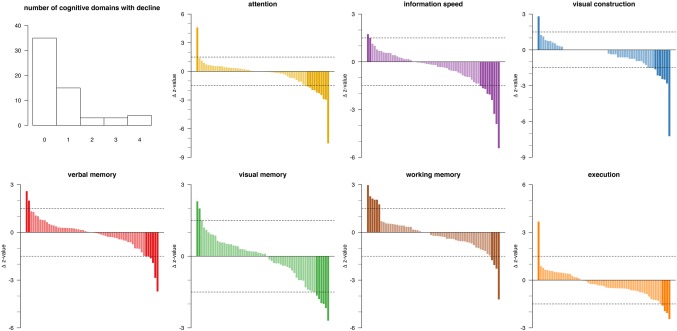
Cognitive alteration per domain between follow‐up and baseline neuropsychological assessment (*n* = 59). Upper‐left: Histogram of number of patients by number of cognitive domains with decline. Bar plots of change in *z*‐sore values in decreasing order of change for each of seven cognitive domains. Each bar represents one patient. Positive values correspond with cognitive improvement; negative values with cognitive decline. The horizontal dotted lines are drawn at +1.5 and −1.5 *SD* as arbitrary thresholds for classification as improvement (far‐left dark‐colored) or decline (far‐right dark‐colored), or unchanged (light‐colored) [Color figure can be viewed at http://www.wileyonlinelibrary.com]

The differences between patients' *z*‐scores at follow‐up and baseline are plotted in Figure 1, indicating that the strict thresholds indeed identified outliers.

**Figure 2:**
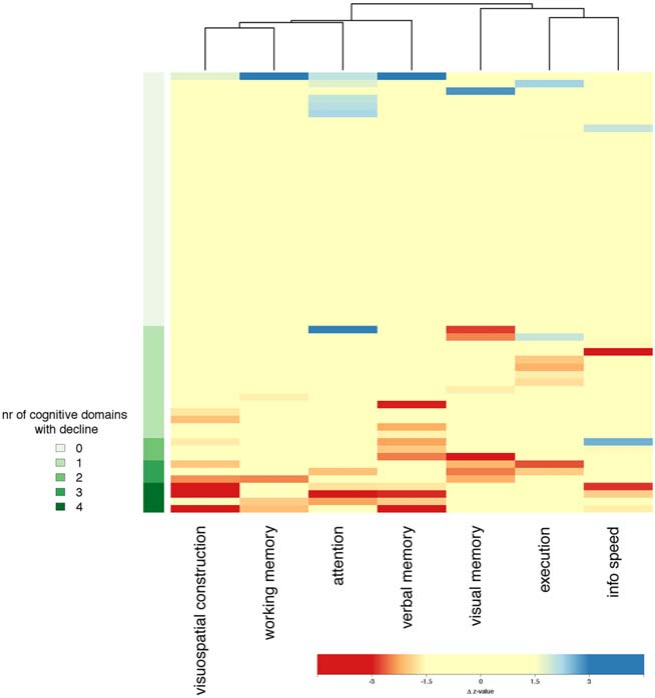
Heat map of cognitive alteration in 59 patients in rows by cognitive domain in columns. Patients are ordered by number of cognitive domains with decline (green color coding on left). Domains are ordered by unsupervised clustering. No clustering profiles are identified. Cognitive decline is shown in shades of red; cognitive improvement in shades of blue; and unchanged cognitive performance in yellow (see bottom color legend) [Color figure can be viewed at http://www.wileyonlinelibrary.com]

Cognitive improvement was observed in one or more domains in 10 (17%) patients. Cognitive decline manifested in one domain in 15 (25%) patients, of whom 4 had normal cognitive performance preoperatively. More than one domain declined in 10 (17%) patients, of whom 5 had normal cognitive performance preoperatively. No clustering profiles of cognitive alteration patterns were identified (Figure 2).

Cognitive improvement was observed for working memory in six (10%) patients, in other domains only incidentally. Cognitive decline was observed in attention in 10 (17%) patients, in information processing speed in nine (15%), in visual construction in seven (12%), in both visual and verbal memory in six (10%), and in both working memory and execution in four (7%).

### 3.3 Language deficits linked to tumor infiltration as positive control

To ensure the robustness of our map comparison, we compared tumor localization maps of the patients with and without a language deficit at presentation (Supporting Information, Movie 1).

All identified regions were in the left hemisphere and were focused on the arcuate fasciculus, the dorsal part of the superior temporal gyrus, the ventral premotor cortex, and the dorsolateral premotor cortex. This confirmation (Banerjee et al., 2015) adds support to the validity of our approach.

### 3.4 Brain regions associated with cognitive decline

**Figure 3:**
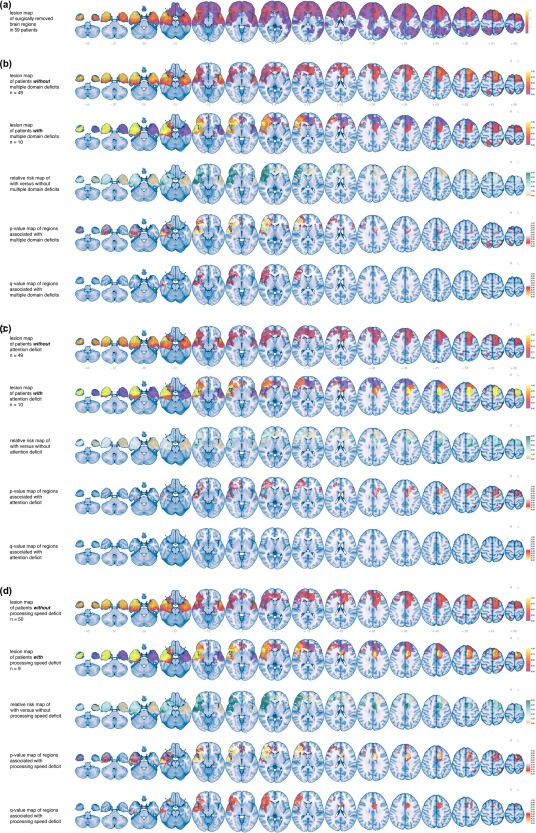
Brain result maps of (a) lesions, here surgically removed regions as resection cavity map, in the study population, demonstrating temporal and frontal preferential locations, with a maximum of 15 patients at a voxel, (b) patients without and with two or more declined cognitive domains, (c) patients without and with an attention decline, and (d) patients without and with a processing speed deficit. For (b–d), rows represent (1) a resection cavity map of the patients without a decline, (2) a resection cavity map of the patients with a decline, (3) a relative risk map with larger differences in resected regions represented by deeper turquoise, (4) a randomized *p* value map with lower *p* values represented by lighter colors, and (5) a *q* value map with lower false discovery rates represented by lighter colors for visual interpretation of other thresholds. All results are superimposed on MNI's standard brain, *z* values are plotted below slices in the first rows. Laterality as indicated [Color figure can be viewed at http://www.wileyonlinelibrary.com]

We then determined the resection map across all 59 patients, corresponding to the preferential locations of gliomas (Figure 3A).

**Figure 4:**
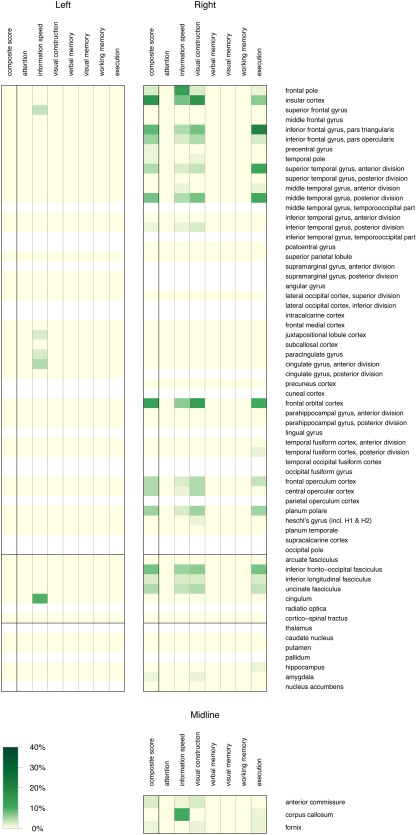
Heat map of percentage of voxels (shades of green) with a *q* value <0.4 in a cognitive domain in columns per anatomical region in rows. The anatomical regions are grouped by laterality and midline structures, and subdivided by horizontal black bars in cortical regions, subcortical white matter pathways, and subcortical grey nuclei. No brain regions were identified for decline in attention, verbal memory, visual memory, and working memory, and therefore, these rows are empty. Absence of information in anatomical regions is demonstrated as white, whereas information of 0% is demonstrated as light yellow [Color figure can be viewed at http://www.wileyonlinelibrary.com]

We aimed to pinpoint the critical brain locations for overall cognitive decline upon surgical removal. Therefore, maps of resected regions were compared between the 10 patients with cognitive decline in more than one domain and the 49 patients with decline in at most one domain (Figure 3B; Supporting Information, Movie 2). All identified regions were located in the right hemisphere, and were centered around the insular cortex, the fronto‐orbital cortex, and the perisylvian cortex, including the inferior frontal gyrus, the superior temporal gyrus, and opercular cortex (Figure 4). This also included peri‐Sylvian white matter structures, such as the inferior fronto‐occipital fasciculus, the uncinate fasciculus, and the inferior longitudinal fasciculus.

As attention was the most‐frequently affected cognitive domain, we proceeded to evaluate the associated brain regions. Resection cavity maps were compared between the 10 patients with an attention decline and the 49 patients with unchanged attention performance (Figure 3C; Supporting Information, Movie 3). No regions were identified with *q* values <40%.

As information processing speed was the next most frequently affected domain, we also compared the maps of resected regions between the nine patients with a reduction in information processing speed and the 50 patients with unchanged information processing (Figure 3D; Supporting Information, Movie 4). Associated brain regions were located in both hemispheres and in the midline, and focused on the right frontal pole, the left cingulum, and the corpus callosum (Figure 4). This also included the right insular cortex, the frontal orbital cortex and peri‐Sylvian white matter structures, such as the inferior fronto‐occipital fasciculus, the uncinate fasciculus, and the inferior longitudinal fasciculus.

The other domains were also analyzed for associated brain regions, although these were involved in fewer patients. Results show that a decline in executive functioning may be particularly associated with surgical removal of the right pars triangularis of the inferior frontal gyrus and the right superior and middle temporal gyrus (Figure 4). A selection of individual tests was also analyzed for the executive functioning domain. Declines in the trail making test A, B, and B‐A, or categoric word fluency score were not associated with surgically removed brain regions at a *q* value <40%.

## 4 DISCUSSION

The main findings of this study are that (a) cognitive alterations after resective surgery of diffuse (nonenhancing) glioma are rather prevalent with 17% of patients declining in more than one domain and 10% improved in at least one domain, (b) attention and information processing speed are the most frequently declined cognitive domains, and (c) the brain regions most vulnerable for cognitive decline after surgery are located in the right hemisphere.

Few studies have reported on cognitive alteration after glioma resection based on pre‐ and postoperative neuropsychological examination (Dallabona et al., 2017; Habets et al., 2014; Mandonnet et al., 2015; Noll et al., 2015; Racine, Li, Molinaro, Butowski, & Berger, 2015; Satoer et al., 2014; Talacchi et al., 2011; Wu et al., 2011). Cognitive decline varied between 24% and 60% in these studies and reported different affected domains. Postoperative testing was within six weeks of surgery in nearly all studies, whereas further cognitive improvement has been observed between three months and one year (Satoer et al., 2014). Furthermore, these studies have included mainly patients with WHO grade IV glioblastoma, in whom tumor mass‐effect on the brain and subsequent surgical relief of compression is likely different from removal of tumor‐infiltrated brain regions in patients with lower grade gliomas. In addition, in these studies, the difference between mean test scores before and after surgery was analyzed for the study group, instead of the frequency of relevant cognitive alterations which, in our opinion, is more informative for patient counseling and clinical decision‐making. Some studies aimed to identify brain locations associated with decline in a specific test, such as in working memory (Teixidor et al., 2007), personality (Campanella, Fabbro, Ius, Shallice, & Skrap, 2015), and mentalization (Duffau, 2014). We decided to analyze composite domain scores rather than individual tests, in line with previous reports (Douw et al., 2009; Klein et al., 2002), because of the presumed higher robustness against noise of individual test scores. This is supported by the finding that the executive functioning domain score identified brain regions that are vulnerable to cognitive decline, whereas individual test scores, that is, the trail‐making test and the categoric word fluency score, did not. Other studies aimed to detect cognitive alterations after glioma surgery in specific brain regions, such as the temporal lobes (Noll et al., 2015), the insula (Wu et al., 2011), or eloquent areas (Mandonnet et al., 2015). Here, we investigated cognitive changes one‐year after surgery, using a combination of tests to cover a variety of domains in a homogeneous population of adults with slow‐growing diffuse gliomas to isolate surgical impact on cognition.

Patients often declined in attention after glioma resection in our series. The attention network is usually divided into an alerting, an executive, and an orienting component. The anatomical distributions of these networks are extensive. The alerting attention network has been localized in frontal and parietal regions with their interconnecting pathways, particularly in the right hemisphere, the executive attention network in the anterior cingulate cortex with connections to the dorsolateral prefrontal cortex, and the orienting network in the thalamus with connections to the superior parietal lobe, temporo‐parietal junction, the superior temporal lobe, and the frontal eye fields (Raz & Buhle, 2006). In a neuro‐imaging meta‐analysis of 173 experiments with normal subjects, activation was observed during attention tasks in bilateral fronto‐parietal pathways, such as the anterior insula, inferior frontal gyrus, dorsolateral prefrontal cortex, dorsal premotor cortex, and intraparietal sulcus (Cieslik, Mueller, Eickhoff, Langner, & Eickhoff, 2015). Lesion mapping studies in patients within two weeks after stroke identified loci associated with declined attention in the peri‐Sylvian cortex, prefrontal and premotor cortical regions, and the thalamus (Corbetta et al., 2015; Murakami et al., 2014; Rinne et al., 2013). Many of these regions were indicated by our *p* value maps, although we were unable to pinpoint associated brain regions in our *q* value maps. The extensiveness of the attention network may explain why this domain was most frequently involved in our study.

Our patients also declined in information processing speed after glioma resection. This domain represents efficiency of overall cognition measured by timing of performances. A decline in information processing speed during aging was associated with global white matter changes (Kuznetsova et al., 2015), and with fractional anisotropy alterations in the corpus callosum, superior longitudinal fasciculus and the inferior fronto‐occipital fasciculus (Kerchner et al., 2012). In stroke, decline of information processing speed was associated with cortical lesions in the left supramarginal and angular gyri and with the superior longitudinal fasciculus (Turken et al., 2008). In cerebral small vessel disease, reduced processing speed was associated with the anterior corpus callosum and right anterior thalamic radiation (Duering et al., 2014, 2011). The connectivity of the mediodorsal nucleus of the thalamus with the frontal and temporal regions (Behrens et al., 2003) may explain why distant lesions can be associated with the same processing speed decline. In our data, similar regions were associated with speed reduction, indicating a disconnection of the projections between the right thalamus and prefrontal and insular cortical regions. The left cingulum was also associated with reduced processing speed in our data. In normal aging, processing speed decline has previously been linked with fractional anisotropy changes of the left cingulum (Sasson, Doniger, Pasternak, Tarrasch, & Assaf, 2013).

Voxel‐based lesion mapping is one established method to relate brain location to brain function (Bates et al., 2003). This has contributed considerably to clinical knowledge of brain function, such as language deficit recovery after stroke (Kümmerer et al., 2013; Price, Seghier, & Leff, 2010). Nevertheless, this technique has limitations, which may result in mislocalization of function. First, mislocalization of functional focal points in the brain may result from spatial bias, introduced in lesion‐deficit mapping by the specific lesion architecture of gliomas. For instance, lesion‐deficit mapping analysis of ischemic lesions may well reflect differences in vascular territories rather than the true functional organization of the brain (Mah, Husain, Rees, & Nachev, 2014). The equivalent in glioma may be the location of origin of different molecular glioma subtypes (Ellingson et al., 2013). Second, more complex and unpredictable interactions between lesions and deficits may arise when tumor is located in many locations or when a function is distributed over many critically functional areas in the brain (Boes et al., 2015; Mah et al., 2014). As a consequence, two patients with the same cognitive deterioration and with damage to the same functional network may have had resections at entirely different brain locations. Or symptoms may arise from anatomically intact brain regions in connection with the lesion‐induced disconnected regions. Third, spatial resolution of lesion mapping may differ from the resolution of functional individual neurons and networks in the brain. Fourth, nonlinear registration is a potential source of mislocalization. This was verified by visual inspection for all patients and the potential error was considered minimal.

In our voxel‐based mapping, we have used resection cavities as lesions to obtain resection cavity maps for lesion‐function mapping. These should be distinguished from resection probability maps that capture the resectability of tumors in patient populations, that have been previously used to estimate the expected residual tumor volume (Mandonnet et al., 2007), to evaluate the potential for brain plasticity (Herbet, Maheu, Costi, Lafargue, & Duffau, 2016; Ius, Angelini, Thiebaut de Schotten, Mandonnet, & Duffau, 2011) to compare resection results between surgical teams (De Witt Hamer et al., 2013).

Our approach has several strengths. First, the study cohort was homogeneous consisting of young adults with a slowly infiltrative diffuse glioma who had standardized imaging and neuropsychological follow‐up. Second, an established voxel‐based lesion‐symptom mapping methodology was applied without presumptions to the association between brain location and function. Third, the assumption‐free randomization test with quantification of false discoveries was used as robust analysis. This is illustrated by the verification of the established brain regions involved in language in our data (Sanai, Mirzadeh, & Berger, 2008; Tate, Herbet, Moritz‐Gasser, Tate, & Duffau, 2014).

This study has some limitations as well. First, the power to detect associations between resected brain and cognitive deterioration follows the preferential locations of glioma, and is therefore, not distributed evenly in the brain. Second, whereas we attempted to isolate the association between resections and cognition, several other factors may have contributed to cognitive alterations, such as psychological burden of disease, early effects of radiotherapy in some patients (Klein, 2012), or surgery‐related ischemia. Cognitive deterioration is associated with late, rather than early, effects of radiotherapy (Douw et al., 2009). New postoperative diffusion‐restricted regions, that is, surgical ischemia, were excluded from our segmentations, but may have contributed to cognitive decline, similar to acquired neurological deficits (Jakola et al., 2014). Third, according to most clinical settings, application of different scanner vendors and field strengths is a commonly known concern, and this could have potentially influenced our voxel‐based lesion mapping analysis.

The clinical relevance of the observed cognitive alterations is difficult to answer because cognition does not correlate with quality of life (Boele et al., 2015; Wolf et al., 2016). Nevertheless, our findings can have clinical implications. Patient counseling should include information on cognitive alterations after glioma resection, in particular for attention and information processing speed, which seems to be at risk after surgery in the right hemisphere. In addition, surgery of the right‐sided peri‐Sylvian cortex and subcortical connections may benefit from mapping of attention tasks. Similarly, surgery of the frontal cortex, cingulum, and the anterior corpus callosum may benefit from mapping of information processing tasks. Intraoperative mapping beyond language and motor functions of specific cognitive tasks has been demonstrated to be feasible (Kinoshita et al., 2016; Wager et al., 2013). Our observations may contribute to identify the patients who can benefit most from these techniques.

## FUNDING

This research is part of the programme Innovative Medical Devices Initiative with project number 10–10400‐96–14003, which is financed by the Netherlands Organisation for Scientific Research (NWO). This research is also supported by a research grant from the Dutch Cancer Society (VU2014–7113).

## CONFLICT OF INTEREST

The authors declare that they have no conflicts of interest to disclose.

## Supporting information

Additional Supporting Information may be found online in the supporting information tab for this article.

Supporting Information

Supporting Information Movie 1

Supporting Information Movie 2

Supporting Information Movie 3

Supporting Information Movie 4
