# Supplementary material for: Linking late cognitive outcome with glioma surgery location using resection cavity maps
Source: Hum Brain Mapp. 2018 Jan 29;39(5):2064–74. doi: 10.1002/hbm.23986 (PMC5947547; doi:10.1002/hbm.23986)
Supplement: Supplementary file 1 — Supporting Information [file HBM-39-2064-s001.docx]

Supplementary Movie 1. Axial sections of tumor-infiltrated brain regions associated with language dysfunction at presentation. The results identify established language regions. Results are superimposed on MNI standard brain template: (1) tumor map of 46 patients without language dysfunction, (2) tumor map of 13 patients with language dysfunction, (3) relative risk map of tumor localization with and without language dysfunction, (4) p-value map of randomization tests, (5) q-value map of false discovery rate. The numbers indicate MNI z-values.

Supplementary Movie 2. Axial sections of resection regions associated with cognitive decline of two or more domains. Results are superimposed on MNI standard brain template: (1) lesion map of surgically-removed regions of 49 patients without decline or decline of one domain, (2) lesion map of 10 patients with cognitive decline in two or more domains, (3) relative risk map of surgical removal with and without cognitive decline in two or more domains, (4) p-value map of randomization tests, (5) q-value map of false discovery rate. The numbers indicate MNI z-values.

Supplementary Movie 3. Axial sections of resection regions associated with attention decline. Results are superimposed on MNI standard brain template: (1) lesion map of surgically-removed regions of 49 patients without decline, (2) lesion map of 10 patients with attention decline, (3) relative risk map of surgical removal with and without attention decline, (4) p-value map of randomization tests, (5) q-value map of false discovery rate. The numbers indicate MNI z-values.

Supplementary Movie 4. Axial sections of resection regions associated with information processing speed decline. Results are superimposed on MNI standard brain template: (1) lesion map of surgically-removed regions of 50 patients without decline, (2) lesion map of 9 patients with information processing speed decline, (3) relative risk map of surgical removal with and without information processing speed decline, (4) p-value map of randomization tests, (5) q-value map of false discovery rate. The numbers indicate MNI z-values.
